# Recurrent meningitis due to *Salmonella arizonae* in an adult with AIDS: case presentation and next-generation sequencing analysis

**DOI:** 10.1128/asmcr.00095-25

**Published:** 2025-08-26

**Authors:** Jairo Lizarazo, Catering Rodriguez, Efrain Montilla-Escudero, Carolina Duarte

**Affiliations:** 1 Hospital Universitario Erasmo Meoz 628866 , Cúcuta, Colombia; 2 Grupo de Microbiología, Instituto Nacional de Salud 67626, Bogotá, Colombia; Rush University Medical Center, Chicago, Illinois, USA

**Keywords:** *Salmonella arizonae*, AIDS-related opportunistic infections, meningitis

## Abstract

**Background:**

Invasive non-typhoidal *Salmonella* disease is a leading cause of morbidity and mortality worldwide, primarily affecting children, the elderly, and immunocompromised adults, particularly those living with HIV. Salmonella meningitis is rare, except in sub-Saharan Africa. *Salmonella arizonae* is associated with reptiles and exceptionally causes meningitis in humans.

**Case Summary:**

We present the case of an adult man living with HIV who presented five episodes of bacterial meningitis due to *S. arizonae* within a period of 8 months. The patient improved after performing cholecystectomy and with optimization of antiretroviral treatment and prolonged antibiotic therapy. Genomic study confirmed that *S. arizonae* is sensitive to antibiotics.

**Conclusion:**

This case illustrates a rare form of *S. arizonae* meningitis. The identity of the etiological agent was confirmed by next-generation sequencing analysis.

## INTRODUCTION

Invasive non-typhoidal *Salmonella* disease is a leading cause of morbidity and mortality worldwide, primarily affecting children, the elderly, and immunocompromised adults, particularly those living with HIV (1). One of the complications of *Salmonella* bacteremia is meningitis, which is rare except in sub-Saharan Africa (2). *Salmonella enterica* subsp. *arizonae* is associated with reptiles and exceptionally causes meningitis in humans (3). We present the case of an adult living with HIV who has had repeated cases of *S. arizonae* meningitis.

## CASE PRESENTATION

We present a case of a 38-year-old man with a history of HIV infection, diagnosed one year earlier, who presented with headache, fever, and vomiting for 24 h of evolution. Previously, within 2 weeks, he had been diagnosed with right-sided bacterial epididymo-orchitis and a urinary tract infection. He received antibiotic treatment with piperacillin/tazobactam 4.5 g IV every 6 h plus clindamycin 600 mg IV for 1 week. In addition, antiretroviral therapy (ART) with abacavir/lamivudine and efavirenz, which he had discontinued 7 months earlier, was restarted; ART was discontinued for economic reasons and due to change of city of residence. No contact with snakes or turtles was reported.

On admission, his vital signs were as follows: blood pressure 107/77 mm Hg, heart rate 115 beats/min, respiratory rate 18 breaths/min, temperature 38°C, BMI 18.6, and Glasgow Coma Scale 15/15. The patient was malnourished but was alert and oriented in space and time. His speech was fluent, and there were no cranial nerve abnormalities. Nuchal rigidity was positive, without meningeal signs. He showed no motor or sensory deficits, and his gait and myotatic reflexes were normal.

Laboratory tests revealed normocytic and normochromic anemia accompanied by leukopenia with lymphopenia. Blood chemistry and urine tests were normal. A chest x-ray revealed no lesions. Cerebrospinal fluid (CSF) analysis was consistent with acute bacterial meningitis, as detailed in Table 1. The treatment administered was ceftriaxone 2 g intravenously every 12 h for 21 days, taking into consideration the blood culture isolation of *Salmonella* group, sensitive to ceftriaxone (Table 1). Rapid improvement in the patient’s condition was observed, and at the conclusion of antibiotic treatment, he was discharged with a prescription of trimethoprim/sulfamethoxazole 160/800 mg orally daily, in addition to ART. The initial simple cranial computer tomography and three repeat scans performed in the following hospitalizations showed marked corticosubcortical cerebral atrophy with frontotemporal predominance and cerebellar atrophy.

**TABLE 1:** Characteristics of the CSF, other laboratory tests, and treatment

Parameters	1st episode day 1	2nd episode day 47	3rd episode day 83	4th episode day 188	5th episode day 260
CSF					
Aspect	Slightly cloudy	Cloudy	Cloudy	Cloudy	Slightly cloudy
Color	Yellowish	Yellowish	Yellowish	Pink	Yellow
Opening pressure (mmH_2_O)	180	170	190	240	240
Glucose (mg/dL)	33	9	7	1	16
Glucorrachia/glucometry	0.27	0.09	0.07	0.01	ND^*c*^
Proteins (mg/dL)	250.86	578	355	120	175
Leukocytes/mm^3^	472	1,817	646	127	1,028
% neutrophils	84	94	79	60	85
% lymphocytes	16	6	21	40	15
Red blood cells	0	0	0	2,000	0
Gram	Negative	Negative	Negative	Negative	Negative
Bacterial culture*^a^*	Negative	Negative	Negative	*S. enterica* ss. *arizonae*	*S. enterica* ss. *arizonae*
Multiplex PCR test (FilmArray meningitis/encephalitis panel*^b^*)	ND	Negative	Negative	Negative	Negative
Indian ink test	Negative	Negative	Negative	Negative	ND
Other laboratory tests					
Plasma leukocytes/mm3	3,210	2,800	3,730	2,210	6,000
Blood cultures*^a^*	*Salmonella* group	Negative	Negative	Negative	*S. enterica* ss. *arizonae*
Urine culture	Negative	ND	ND	Negative	Negative
Coproculture	ND	ND	ND	Negative	Negative
Bile culture	ND	ND	ND	ND	Negative
Multiplex PCR test (FilmArray gastrointestinal panel)	ND	ND	ND	ND	*Shigella*/EIEC^*d*^
CD4+ cells	ND	ND	10	18	ND
Viral load (copies/mL)	ND	ND	8,734	107,000	ND
Toxoplasma IgG (U/mL)	0.130	0.130	ND	5.13	ND
Cytomegalovirus IgM (U/mL)	ND	0.175	ND	0.140	ND
Cytomegalovirus IgG (U/mL)	ND	243.3	ND	223.3	ND
Treatment					
Antibiotic, duration of treatment (days)	Ceftriaxone, 21	Ceftriaxone, 21	Ceftriaxone, 42	Meropenem, 42	Meropenem, 42

^*a*^ For bacterial identification, a Vitek 2 system (bioMérieux, France) was used.

^*b*^ The panel does not identify *Salmonella*.

^*c*^ ND, not done.

^*d*^ EIEC, enteroinvasive *E. coli*.

In the following months, the patient experienced four additional admissions for similar clinical pictures, two of which were characterized by mild alterations of consciousness (Table 1). During his second and fourth hospitalizations, he experienced generalized tonic-clonic seizures with loss of consciousness, which were treated with phenytoin; the electroencephalogram showed a slight abnormality due to the presence of slow-wave bursts in the frontal regions.

In the first three hospitalizations, antibiotic treatment was started at least one day before the diagnostic lumbar puncture was performed. In the fourth and fifth hospitalizations, a change in treatment was implemented, replacing ceftriaxone with meropenem 1 g IV every 12 h for 42 days (Table 1).

In the third episode, the patient presented herpes simplex labialis, which was treated with topical acyclovir. In the fourth hospitalization, a change in ART was implemented, considering the low response (Table 1), and treatment with dolutegravir and emtricitabine/tenofovir was initiated. In the same hospitalization, a decrease in bilateral visual acuity was observed, with predominance in the left eye, and cytomegalovirus retinitis was diagnosed. Treatment consisted of intravenous ganciclovir. In the following hospitalization, treatment was completed with the application of intravitreal ganciclovir in the left eye.

At the last hospitalization, hepatobiliary ultrasound showed cholecystitis and cholelithiasis; the patient had not reported pain, and abdominal palpation was normal. A laparoscopic cholecystectomy was performed without complications, and the bile culture was negative.

The patient was discharged from his fifth hospitalization without neurological deficit. He was prescribed continued antiretroviral and anticonvulsant therapy, as well as trimethoprim/sulfamethoxazole, fluconazole, and nutritional support. He did not develop meningitis for the next 3 years and finally died of unspecified lymphoma.

### Study of the isolations

Three isolates were obtained in different episodes (the first, fourth, and fifth), one from blood culture and two from CSF, which were identified as *Salmonella* group (the first) and as *S. arizonae* (the others) by the Vitek 2 system at the Hospital Universitario Erasmo Meoz (Table 1), all of them showed an identical susceptibility profile based on the minimal inhibitory concentration (MIC) (Table 2). These isolates were sent to the Instituto Nacional de Salud to confirm the serotype.

**TABLE 2:** MIC profile of *S. arizonae* isolates from CSF and blood by hospitalization episode^*a*^

MIC/source and isolate	*Salmonella* group blood	*S. arizonae* CSF	*S*. *arizonae* CSF
Hospitalization	1st	4th	5th
Amikacin	NT^*b*^	NT	NT
Ampicillin	NT	NT	≤2
Ampicillin-sulbactam	≤2	≤2	≤2
Aztreonam	NT	NT	≤1
Cefepime	≤1	≤1	≤1
Cefoxitin	NT	NT	NT
Ceftazidime	≤1	≤1	≤1
Ceftriaxone	≤1	≤1	≤1
Ciprofloxacin	≤0.25	≤0.25	≤0.25
Colistin	≤0.5	≤0.5	NT
Doripenem	≤0.12	≤0.12	NT
Ertapenem	≤0.5	≤0.5	≤0.5
Gentamicin	NT	NT	NT
Imipenem	≤0.25	≤0.25	NT
Meropenem	≤0.25	≤0.25	≤0.25
Piperacillin-tazobactam	≤4	≤4	≤4
Tigecycline	≤0.5	≤0.5	≤0.5
Trimethoprim-sulfamethoxazole	NT	NT	≤2

^*a*^ The AST test platform used was Vitek 2 AST-N402 card and the clinical breakpoints according to document M-100 CLSI 2019 version. Values displayed are minimal inhibitory concentrations (mg/L).

^*b*^ NT, not tested.

The conventional WHO 2021 serotyping test (4) characterized the microorganism as Salmonella “O:48 (Y)” with flagellar antigens “z4 (+), z24 (+), z23 (−), z32 (−),” suggesting that the possible serotype and subspecies were *Salmonella enterica* subsp. *enterica* serovar Djakarta, *S*. *arizonae* (IIIa) or *Salmonella enterica* subsp. *diarizonae* (IIIb), and thus genetic sequencing was considered to specify the serotype.

**Fig 1:**
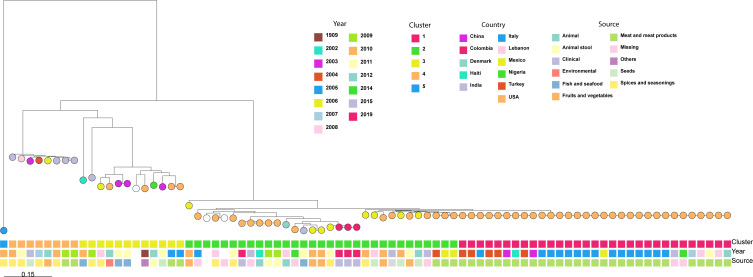
Maximum likelihood phylogenetic tree of *S. enterica* subsp. *arizonae*. The phylogenetic tree was constructed based on a core genome SNP analysis and includes three Colombian isolates obtained from a single patient, along with publicly available genomes from NCBI. The tree illustrates the evolutionary relationships among *S. enterica* subsp. *arizonae* isolates, annotated by genetic cluster, year of isolation, source, and country of origin. Five distinct genetic clusters (Clusters 1–5) were identified and are color-coded. The three Colombian isolates grouped tightly within the same clade and showed no SNP differences among them, indicating a clonal origin and supporting the hypothesis of a persistent infection from a single source. In contrast, comparisons with international isolates associated with foodborne outbreaks—such as dried oregano (Mexico), fresh spinach (Mexico), and vannamei shrimp (India)—revealed high genetic distances (greater than 1,485 SNPs), suggesting no recent epidemiological link or common source with these strains. Overall, the tree highlights the genetic diversity of *S. enterica* subsp. *arizonae*, which is associated with a variety of sources, including clinical, environmental, and food-related origins. The presence of strains linked to spices, seafood, and fresh produce underscores the potential of this subspecies to persist across different ecological niches and be transmitted through multiple food chains.

Genomic analysis of the three isolates was performed on the MiSeq System (Illumina) using paired 2 × 200 bp reads. The raw data and assemblies obtained from sequencing were used for *in silico* serotyping, confirming *S. arizonae* serovar 48:z4,z24 with SeqSero2 (https://github.com/denglab/SeqSero2) and *S. arizonae* IIIa serovar Y:z4,z24:- with Salmonella In Silico Typing Resource (SISTR) (https://github.com/phac-nml/sistr_cmd). The type of sequence obtained was ST: 2765, which is not specific to this species but has been isolated from *S. arizonae* in chickens and shrimp (https://enterobase.warwick.ac.uk/) (Fig. 1).

The *in silico* susceptibility profile presented the same behavior as the CLSI phenotypic sensitivity (5); however, resistance to elfamycin, an antibiotic used as a growth promoter in chickens, was detected.

The presence of pathogenicity island 9 was confirmed in all three isolates. This island, which is associated with virulence factors, encodes a type I secretion system and a function found in *S*. Typhi. This function is responsible for adhesion induced under conditions of high osmolarity in culture (6).

## DISCUSSION

*Salmonella* is a Gram-negative bacillus with more than 2,500 different serotypes. The current taxonomic classification of the genus *Salmonella* includes two species: *Salmonella enterica* and *Salmonella bongori. S. enterica* comprises six subspecies: *arizonae*, *diarizonae*, *enterica*, *houtenae*, *indica,* and *salame* (7). From the clinical point of view, *Salmonellae* are classified into two groups: those that produce typhoid fever and those that do not (8). *Salmonella* meningitis is rare in developed countries and usually affects children under 5 years of age and immunocompromised adults (9).

Our patient was severely immunosuppressed and had a poor nutritional status that favored *Salmonella* infection. The episodes of acute meningitis presented with the classic clinical presentation of headache, fever, nuchal rigidity, and altered consciousness. CSF findings were characteristic: polymorphonuclear pleocytosis, elevated protein, and low glucose; Gram staining was not helpful, and the absence of bacterial growth in the first three CSF cultures may be attributed to prior antibiotic use. The *S. arizonae* isolates were all sensitive to ceftriaxone and meropenem, the antibiotics used in treatment (Table 2).

Despite prolonged antibiotic treatment, relapse of *Salmonella* meningitis is frequent (9). Recurrent *Salmonella* bacteremia is one of the AIDS-defining entities (10).

Asymptomatic carriers of *S.* Typhi have cholelithiasis in 90% of cases. Likewise, this bacterium has been isolated in bile and vesicular tissue (11). However, the carrier state of non-typhoidal salmonellae is less frequent and is estimated to occur in 0.1% of patients (12). Recurrence of sepsis due to *S*. *arizonae* has been reported in immunocompromised patients after 1 year (13). It is believed that gallstones and chronic cholecystitis facilitate the persistence of the bacteria in this organ (11).

*S. arizonae* is an opportunistic pathogen in immunocompromised patients and is commonly found in reptiles. It has been reported in patients who have ingested rattlesnake dust; also, it has been seen in individuals who keep snakes or turtles as pets (3). There have been only three reported cases of *S. arizona* meningitis, all of them in children under 3 months of age (14, 15).

*S. arizonae* has 100 serovars and has a wide range of hosts such as chickens, ducks, pheasants, and turkeys; in the latter, it has a high mortality (3.5%–90%). Arizonosis is more frequent in North America, and these hosts are infected with food contaminated with reptile feces; therefore, it cannot be ruled out that the patient’s infection may have come from the consumption of birds or eggs and that it can be correlated with some of the genomic findings (16).

The favorable patient outcome can be attributed to three main factors: cholecystectomy, prolonged administration of antibiotics, and optimization of HIV infection control.

Finally, it is important to consider that non-typhoidal *Salmonella* can cause meningitis in adult patients living with HIV, necessitating protracted antibiotic treatment. In the event of recurrence, the presence of a reservoir such as the gallbladder should be considered, along with the possibility of cholecystectomy if cholelithiasis and cholecystitis are present.

## Data Availability

All sequence data associated with this analysis have been deposited in the NCBI database under Sequence Read Archive accession numbers SRR22742368, SRR22742369, and SRR22742370.
